# Utilizing Morphological and Physiological Parameters of *Lemna minor* for Assessing Tetracyclines’ Removal

**DOI:** 10.3390/molecules29163971

**Published:** 2024-08-22

**Authors:** Łukasz Sikorski, Agnieszka Bęś, Kazimierz Warmiński, Wojciech Truszkowski, Przemysław Kowal

**Affiliations:** 1 Department of Chemistry, Faculty of Agriculture and Forestry, University of Warmia and Mazury in Olsztyn, Pl. Łódzki 4, 10-727 Olsztyn, Poland; agnieszka.bes@uwm.edu.pl (A.B.); kazimerz.warminski@uwm.edu.pl (K.W.); 2 Department of Agrotechnology and Agribusiness, Faculty of Agriculture and Forestry, University of Warmia and Mazury in Olsztyn, M. Oczapowskiego 8, 10-719 Olsztyn, Poland; wojciech.truszkowski@uwm.edu.pl; 3 Department of Sanitary Engineering, Faculty of Civil and Environmental Engineering, Gdansk University of Technology, Narutowicza 11/12, 80-233 Gdansk, Poland; przkowal@pg.edu.pl

**Keywords:** antibiotics, tetracycline, minocycline, aquatic plant, biosorption, phytotoxicity, photosynthesis

## Abstract

Antibiotics with significant environmental toxicity, e.g., tetracyclines (TCs), are often used in large quantities worldwide, with 50–80% of the applied dose ending up in the environment. This study aimed to investigate the effects of exposure to tetracycline hydrochloride (TC) and minocycline hydrochloride (MIN) on *L. minor*. Our research evaluated the phytotoxicity of the TCs by analyzing plant growth and biomass and evaluating assimilation pigment levels and fluorescence. The research was extended with the ability potential of duckweed as a tool for removing TCs from water/wastewater. The results demonstrated that both TCs influenced Ir, Iy, biomass, and photosynthetic efficiency. The uptake of TC and MIN by duckweed was proportional to the concentration in the growth medium. The TC was absorbed more readily, reaching up to 8.09 mg × g^−1^ of dry weight (DW) at the highest concentration (19.2 mg × L^−1^), while MIN reached 6.01 mg × g^−1^ of DW. As indicated, the consequences of the influence of TC on plants were slightly smaller, in comparison to MIN, while the plants could biosorb this drug, even at the lowest tested concentration. This study has shown that using plants for drug biosorption can be an effective standalone or complementary method for water and wastewater treatment.

## 1. Introduction

The global scarcity of fresh water has emerged as a pressing issue, exacerbated by ongoing degradation in its quality and availability for both domestic and industrial use, largely due to pollution. This decline is attributed to a multitude of sources, including various industries, agricultural and mining practices, and municipal wastewater [1,2,3]. As a consequence of these activities, the environment can be contaminated with various xenobiotics, including heavy metals [4,5] and plant protection products [6,7,8], but also drugs. Drugs, including antibiotics, constitute a significant class of xenobiotics. Antibiotics are extensively utilized in various sectors of modern society, such as human and veterinary medicine, agriculture, livestock farming, fish farming, and aquaculture [9]. According to the World Health Organization (WHO), the animal sector accounts for roughly 80% of the total consumption of medically significant antibiotics [10]. Annually, the global consumption of antibiotics exceeds 100,000 tons, highlighting the magnitude of this issue [11]. Veterinary medicines are used indiscriminately (treating an entire herd rather than a single animal) and excreted unchanged (some antibiotics and deworming agents). This ultimately becomes an uncontrolled source of environmental pollution and contributes to imbalances in ecosystems. The use of ever-increasing doses of long-known antibiotics due to increasing antibiotic resistance, the introduction of new chemotherapeutics to treat humans and animals, and the increase in the number of multisite industrial farms result in increased environmental pollution by these compounds and their metabolites [12]. The potential significant ecotoxicity of drugs also results from their direct use in the environment, e.g., in aquaculture and antibiotic baths. Antibiotics with significant environmental toxicity, e.g., tetracyclines, are often used in large quantities worldwide, with 50–80% of the applied dose ending up in the environment [13]. From calculations obtained from 41 countries, the consumption of antibiotics in livestock in 2017 was 93,309 tons. Meanwhile, a prognosis using multivariate regression models shows that global sales in 2030 will increase by 11.5% to 104,079 tons [14]. Tetracyclines (TCs), including tetracycline (TC) and minocycline (MIN), are among the most widely used drugs in human and veterinary medicine due to their broad spectrum of action and low cost compared to other antibiotics [15,16,17]. The bioavailability of tetracycline is less than 40% via intramuscular injection, 100% intravenously, and 60–80% orally [18]. And about 50–90% of the parent compound, in a biologically active form [18], is excreted in feces and urine into the environment [19,20,21]. The concentration of tetracycline in pig manure is 23 µg × g^−1^ [22], while the maximum concentration of minocycline in pig feces after injection can be as high as 36.5 µg × g^−1^ [23]. Tetracyclines are water-soluble and have a long half-life span in the environment [24,25]. Ultimately, slurry and slurry from farm animals are transferred to agricultural fields, where the antibiotics and their metabolites contained in them are transported along with precipitation to ground and surface waters and are taken up by crops and aquatic plants [8,26] thus entering into the trophic chain. The global daily intake of tetracycline by humans has been estimated to be approximately 23 kg [27]. As a result, the tetracycline (TC) levels in raw influent and treated effluent ranged from 1.24 to 12.34 μg × L^−1^ and 0.12 to 1.53 μg × L^−1^, respectively [28]. Systematically, MIN also ends up in effluent. Already in 2003, it was estimated that hospitals and households in Germany consumed 815 kg of MIN per year, of which 10% of this amount went to wastewater [29].

While wastewater treatment plants (WWTPs) are efficient in removing organic matter and various mineral and organic compounds, they typically struggle to effectively eliminate pharmaceutical residues from wastewater. The WWTPs primarily target substances outlined in wastewater discharge regulations, leaving pharmaceutical compounds largely unaffected by conventional treatment methods [30]. Consequently, recycled wastewater often retains traces of pharmaceuticals. Various techniques and technologies are used to treat TC wastewater, including adsorption, coagulation, ozonation, electrolysis, ion exchange, or membrane separation processes [31]. Over the past several years, numerous attempts have been made to find the most suitable and safe methods for pharmaceutical removal, including physical and chemical techniques, as well as combining these techniques, e.g., the peroxymonosulfate-assisted photocatalytic degradation of antibiotic norfloxacin by a calcium-based Ag_3_PO_4_ composite or the simultaneous degradation of amoxicillin and norfloxacin by TiO_2_@nZVI composites coupled with persulfate [32,33]. Biological methods are also used to remove drugs. Among these methods, biological processes were often favored due to their low impact on the environment and cost-effectiveness [34,35]. In response, researchers are exploring alternative strategies, particularly biological methods, to mitigate the detrimental impacts of pharmaceuticals on aquatic ecosystems and the resident flora. Aquatic plants play critical roles in these ecosystems as habitats for organisms, sources of oxygen, and primary producers [36]. One such method involves biosorption, a physicochemical process in which biological materials remove contaminants from a solution. Biosorption can occur actively or passively, with pollutants binding to cell structures. Through passive transport facilitated by the transpiration stream, 43 [37]. Biosorption encompasses the entirety of interactions between an adsorbate and a biological matrix. This phenomenon can manifest in dead cell and tissue fragments and be facilitated by living organisms, in which adsorption occurs via surface complexation. The adsorbate adheres to cell walls and external cell layers in complexation occurring on the surface. This initial step of adsorption is both reversible and faster than the broader and more intricate bioaccumulation mechanism [38].

*L. minor* was chosen as a biodegradation agent, as it is applied as an indicator species in environmental risk analysis; thus, it could also be used to assess the toxicity of medicines in water. This study aimed to investigate the effects of exposure to two antibiotics, TC and MIN, which are widely used drugs belonging to the first-generation tetracyclines, naturally occurring and second-generation, semi-synthetic [17], respectively, on *L. minor*. Common duckweed plays a crucial role in freshwater ecosystems as an essential component of the food chain [39,40]. Traditional toxicity assessments primarily focus on common duckweed’s growth parameters, such as growth rate and yield. However, these assessments often overlook the biochemical aspects of plant productivity and specific response mechanisms to toxic substances [41], which are addressed in this study. Our research evaluated the phytotoxicity of the studied antimicrobials, not only through morphological parameters but also by analyzing the physiological parameters of *L. minor*. Antibiotics threaten human health and ecological security due to their toxicity and role in promoting resistant bacteria. They also accumulate in organisms, affecting their survival, growth, and reproduction. Developing alternative strategies and technologies to prevent and remove antibiotic residues from the environment is essential. The aim was to investigate the potential of duckweed as a tool for removing these pharmaceuticals from wastewater and water through the use of model samples contaminated with the tested TCs.

## 2. Results

### 2.1. Effect of TCs on the Ir and Iy of Common Duckweed

**Figure 1:**
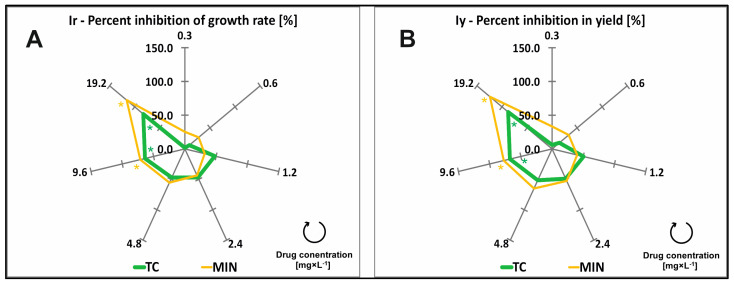
Percent inhibition of growth rate (Ir) (**A**), percent reduction in yield (Iy) (**B**) of common duckweed (*L. minor*) exposed to different concentrations (0–19.2 mg × L^−1^) of tetracycline hydrochloride (TC) and minocycline hydrochloride (MIN). Data points represent the mean ± SD, n = 6. * Values differ significantly from the control at *p* < 0.01.

The impact of increasing TC and MIN concentrations (0; 0.3; 0.6; 1.2; 2.4; 4.8; 9.6; and 19.2 mg × L^−1^) in the growth medium on the tetracycline (TC) and minocycline (MIN) in common duckweed plants exposed to TCs, the percent inhibition of growth rate (Ir), and the percent reduction in yield (Iy) were evaluated based on the number of fronds and frond area. The analyzed concentrations of TCs induced significant changes in the Ir and Iy of common duckweed (Table 1). After 7 days of exposure to the lowest concentration (0.3 mg × L^−1^) of TC and MIN, the Ir of duckweed plants was determined at 1.65 and 25.27%, respectively. These changes were not significant. A significant effect on Ir was noted only from the concentration of 9.6 mg × L^−1^, both for TC and MIN. The highest concentration of the tested drugs (19.2 mg × L^−1^) increased Ir values by 82.87 and 116.00% in plants exposed to TC and MIN (Figure 1A, Table A1).

The Iy of common duckweed plants increased proportionally with a rise in TC concentrations in the medium. At the lowest concentration of TCs (0.3 mg × L^−1^), the most toxic was MIN (Iy = 33.37%). Meanwhile, the Iy for TC was only 5.96%. At the highest concentration (19.2 mg × L^−1^), the Iy for TC was 87.74%, and for MIN, it was higher by 36.26 pp. (Iy = 124%) (Figure 1B, Table A1).

The predicted toxic units were calculated based on TC content and EC values. The 7-day chronic toxicity test revealed that TC and MIN were toxic for common duckweed and increased the values of Ir and Iy when applied at concentrations higher than EC_20_ = 0.69 mg × L^−1^ and EC_20_ = 0.22 mg × L^−1^, respectively. Tetracycline and MIN applied at concentrations of 3.46 mg × L^−1^ and 2.78 mg × L^−1^ (EC_50_), respectively, increased the Ir and Iy of common duckweed by 50% (Table 2).

### 2.2. Fresh Mass and Dry Matter Content

**Figure 2:**
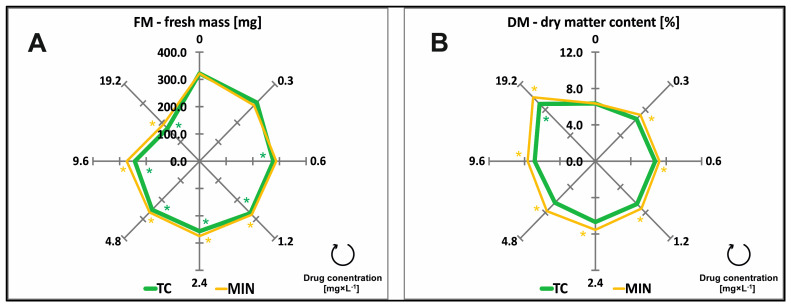
Fresh mass (FM) (**A**) of 100 fronds and dry matter content (DM) (**B**) of common duckweed (*L. minor*) exposed to different concentrations (0–19.2 mg × L^−1^) of tetracycline hydrochloride (TC) and minocycline hydrochloride (MIN). Data points represent the mean ± SD, n = 6. * Values differ significantly from the control at *p* < 0.01.

A fresh mass of 100 duckweed plants (FM) was analyzed after 7 days of exposure to TCs. The type and concentrations of antibiotics significantly influenced the FM of duckweed (Table 1). The FM of control plants was 321.70 mg. Fresh mass insignificantly decreased already in response to the lowest antibiotic concentration (0.3 mg × L^−1^), from 0.6 mg × L^−1^ concentration, FM changes were already significant for TC, and from a concentration of 1.2 mg × L^−1^ for both tested drugs (Figure 2A, Table A1).

Fresh mass continued to decrease higher concentrations of all TCs, for TC and MIN, and was determined at 171.01 and 191.24 mg under exposure to the highest (19.2 mg × L^−1^) concentration, respectively (Figure 2A). Tetracycline was the more toxic drug that decreased FM by 20% (EC_20_) when applied at a concentration of 2.41 mg × L^−1^, whereas the same effective concentration for MIN was almost five times higher (Table 2).

The dry matter content (DM) of control plants was 6.35%. The type and concentrations of antibiotics significantly influenced the DM of duckweed (Table 1). Increasing concentrations of TC and MIN promoted tissue dehydration and led to a steady increase in DM values (Table 1). A significant increase in plant DM was observed under exposure to MIN, starting from the lowest drug concentration (0.3 mg × L^−1^), to achieve the value of 9.94% at the highest MIN concentration (19.2 mg × L^−1^). The average DM of plants treated with TC reached 6.98% (Figure 2B). Minocycline was over 3.5 times more phytotoxic to plant DM than TC, which corresponds to EC_20_ (Table 2).

### 2.3. Chlorophylls, Carotenoids, Phaeophytinization, and Fluorescence

**Figure 3:**
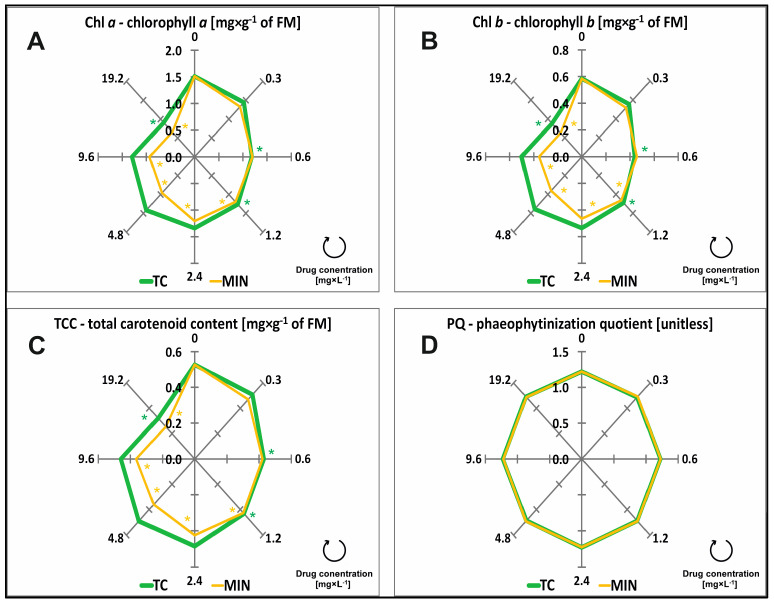
Content of chlorophyll *a* (Chl *a*) (**A**), chlorophyll *b* (Chl *b*) (**B**), total carotenoid content (TCC) (**C**), and the phaeophytinization quotient (PQ) (**D**) of common duckweed (*L. minor*) exposed to different concentrations (0–19.2 mg × L^−1^) of tetracycline hydrochloride (TC) and minocycline hydrochloride (MIN). Data points represent the mean ± SD, n = 6. * Values differ significantly from the control at *p* < 0.01.

The leaves of the control plants (grown on a medium without antibiotics) contained 1.51 mg × g^−1^ chlorophyll *a* (Chl *a*) and 0.58 mg × g^−1^ chlorophyll *b* (Chl *b*), and their total carotenoid content (TCC) was determined at 0.53 mg × g^−1^ of FM (Figure 3A–C). The content of all photosynthetic pigments was significantly modified by the type and concentration of tested drugs (Table 1).

The content of Chl *a*, Chl *b*, and TCC significantly decreased already in response to a 1.2 mg × L^−1^ concentration of TC and MIN. Minocycline in concentrations of 2.4, 4.8, and 9.6 mg × L^−1^ was more toxic than TC and decreased the content of Chl *a*, Chl *b*, and TCC by 19.99%, 35.46%, and 36%, respectively. When the tested drugs were applied at the highest concentration (19.2 mg × L^−1^), the greatest (significant) reduction in the content of Chl *a*, Chl *b*, and TCC in 55% on average was observed under the influence of MIN. Tetracycline was the least toxic, and the highest concentration of MIN (19.2 mg × L^−1^) decreased the content of Chl *a*, Chl *b*, and TCC by 38.94% on average (Figure 3A–C, Table A1). The chlorophyll level in plant tissues was reduced by 20% (EC_20_) in the presence of 0.67 and 0.60 mg × L^−1^ of TC and MIN, respectively, in the growth medium. In turn, the content of Chl *b* decreased by 20% (EC_20_) under exposure to TC and MIN at 0.63 and 0.57 mg × L^−1^, respectively. Minocycline reduced TCC by 20% (EC_20_) at a concentration of 2.43 mg × L^−1^, while TC at a concentration almost 6.5 times higher (Table 2). None of the tested TCs influenced phaeophytinization quotient (PQ) values. PQ reached 1.23 on average in all samples (Table 1, Figure 3D, Table A1).

**Figure 4:**
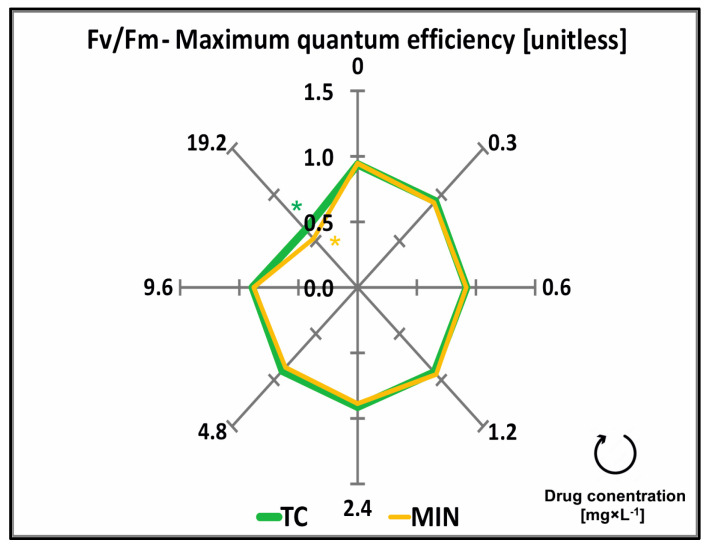
Maximum quantum efficiency (Fv/Fm) of common duckweed (*L. minor*) exposed to different concentrations (0–19.2 mg × L^−1^) of tetracycline hydrochloride (TC) and minocycline hydrochloride (MIN). Data points represent the mean ± SD, n = 6. * Values differ significantly from the control at *p* < 0.01.

The maximum quantum efficiency (Fv/Fm) ratio was affected by both the type and the concentration of the applied antibiotic (Table 1). Duckweed plants exposed to TC and MIN in concentrations of 0–9.6 mg × L^−1^ were characterized by a 0.91 Fv/Fm ratio. At the highest concentration of TC and MIN (19.2 mg × L^−1^), the Fv/Fm ratio was 33.7% and 44.3%, significantly lower, respectively, than in the control (Figure 4, Table A1).

### 2.4. Antibiotic Biosorption—Results

**Figure 5:**
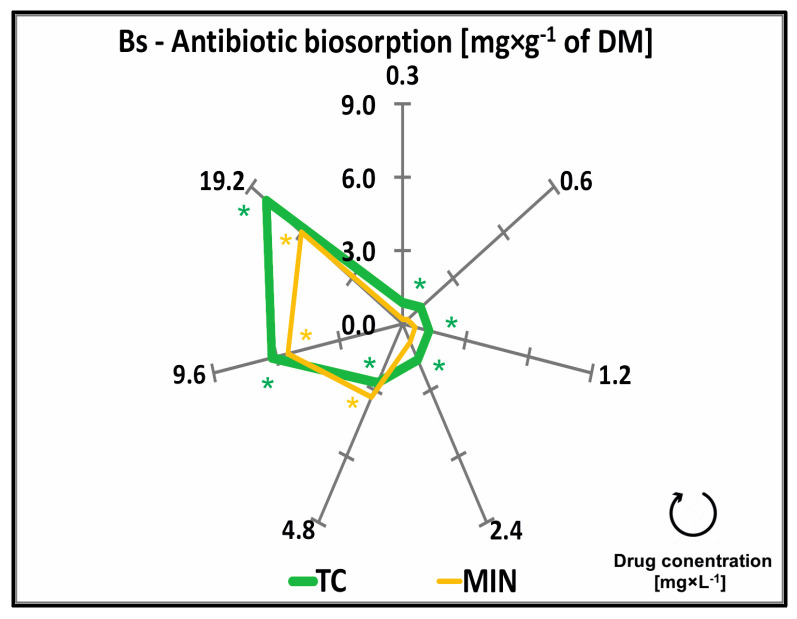
Antibiotic biosorption (Bs) by common duckweed (*L. minor*) exposed to different concentrations (0–19.2 mg × L^−1^) of tetracycline hydrochloride (TC) and minocycline hydrochloride (MIN). Data points represent the mean ± SD, n = 6. * Values differ significantly from the control at *p* < 0.01.

The antibiotic biosorption (Bs) of TC and MIN by common duckweed during the 7-day chronic toxicity test was directly proportional to drug concentrations in the growth medium. Tetracycline was the most readily absorbed among the tested antibiotics. Its content in common duckweed tissues was 0.87 mg × g^−1^ DW (statistically significant) at the lowest concentration of this drug (0.3 mg × L^−1^), and it reached 8.09 mg × g^−1^ DW at its highest concentration (19.2 mg × L^−1^). The Bs of MIN was somewhat lower, and it was determined at 0.20 (statistically insignificant) and 6.01 mg × g^−1^ DW (statistically significant) in plants exposed to 0.3 and 19.2 mg × L^−1^ of MIN, respectively (Figure 5, Table A1).

## 3. Discussion

This study introduced a biological sorption model utilizing *L. minor* plants for water and wastewater treatment. The biosorption findings of various pharmaceuticals, coupled with toxicity assessments following ISO [40] and OECD guidelines [42], indicate its potential as a viable complement or alternative to conventional wastewater treatment techniques. As per the above guidelines, toxicity evaluation should hinge on the morphological traits of the plants. Our investigation identified MIN as exhibiting greater phytotoxicity toward *L. minor* compared to TC among the tested drugs. Plant growth and the development of morphological traits in plants are often inhibited under exposure to most pharmaceuticals that are present in soil and water [8,26,43,44,45,46]. The present study analyzed the effects of two TCs, TC and MIN, on Ir, Iy, Chl *a*, Chl *b*, TCC, PQ, and Fv/Fm and tested drug biosorption (Bs) by *L. minor* plants. Already at the lowest concentration tested, although not significantly, the effect of each drug used inhibited the growth rates of the aquatic plants tested (Table 1). In the case of TC and MIN, no growth stimulation was observed. Low concentrations of pharmaceuticals in soil can have a beneficial impact on plants, potentially stimulating their growth—a phenomenon known as hormesis occurring at low drug concentrations [47]. However, the influence of pharmaceuticals on plants is not solely determined by their concentration, host species, or exposure duration but also by the drug’s form. In a study by [46], a 1.25 mM dose of soluble ciprofloxacin proved lethal for duckweed (resulting in a 100% decrease in Ir and Iy), whereas insoluble ciprofloxacin did not elicit such toxic effects, even at the highest dosage (40 mM). In the 7-day chronic toxicity test, it was observed that TC and MIN exhibited toxicity toward common duckweed, leading to elevated Ir and Iy values when administered at concentrations exceeding EC_20_ = 0.69 mg × L^−1^ and EC_20_ = 0.22 mg × L^−1^, respectively. Plants exposed to antibiotics exhibit impaired growth due to various internal reactions. Conserved target sites for antibiotics stem from the bacterial ancestry of plastid organelles. Gyrase-specific drugs like coumarin novobiocin inhibit chloroplast transcription. Antibiotics such as tetracycline, macrolides, lincosamides, aminoglycosides, and pleuromutilins disrupt plastid translation and transcription. Tetracyclines primarily cause phytotoxicity by chelating metal nutrients in growth solutions rather than directly interfering with plastid ribosomes [48]. The plant growth inhibition results in a reduction in FM (total wet weight) of *L. minor*. The FM decreased significantly for TC and MIN, from a concentration of 1.2 mg × L^−1^ in the medium (Figure 2A). Tetracycline was the more toxic drug that decreased FM by 20% (EC_20_) when applied at a concentration of 2.41 mg × L^−1^, whereas the same effective concentration for MIN was almost five times higher (Table 2). Fresh plant mass is practically the only feature; it reacted less sensitively to contamination of the medium with the contaminants tested. Fresh plant mass is virtually the only feature; it was less sensitive when the plants were exposed to contaminated MIN medium. However, FM changes do not fully reflect the condition of the seedlings, including the disintegration of their tissues. As indicated by the literature data, the yield biomass of *Lemna aequinoctialis* is reduced by 45% due to 10 mg × L^−1^ streptomycin. As indicated by the authors, in all configurations of *L. aequinoctialis*, no chlorosis was observed on the leaves, but samples with higher concentrations of 15–20 mg × L^−1^ streptomycin clearly showed leaf necrosis [49]. Krupka et al. [50] indicate that necrotic changes in plants are caused by the death of mitochondria, the reduction of which by 93% is the result of exposure of *L. minor* to 10 mM of TC. However, TC is not the sole pharmaceutical capable of impeding the growth of common duckweed. It was found that the Ir and Iy of common duckweed are inhibited by quinolones. In research on *L. minor*, concentrations of 12.8 mg × L^−1^ of quinolones are not lethal to indicator plants. However, it inhibits their growth by 82% for levofloxacin and by 62% on average for nalidixic acid, pefloxacin, and moxifloxacin [26]. Similarly, various chemical compounds, akin to many tested TC concentrations, can induce toxic effects on common duckweed. For instance, sodium chloride and glyphosate herbicides have been shown to hinder plant growth, reduce FM, and increase DM [51,52], while polyethylene microbeads have been observed to impede root growth and diminish the viability of root cells [53]. Plants exposed to drugs in water not only show changes in growth and biomass but also clearly show disruptions in water uptake, as indexed by plant DM. Increasing concentrations of TC and MIN promoted tissue dehydration and led to a steady increase in DM values (Figure 2). Xenobiotics, especially in high concentrations in water, are manifested in the form of hypertonic and hyperosmotic stress, which results in the dehydration of *L. minor* tissues and an increase in DM [8]. When the osmotic pressure in plants decreases, they are unable to take up the water, which results in stomata closing, a drop in turgor pressure, membrane disintegration, dehydration of tissues, and a systematic increase in DM. The effects mentioned above of plant exposure to pharmaceuticals are not the only costs these organisms incur to compensate for adaptation costs. In our study, the content of all photosynthetic pigments was significantly modified by the type and concentration of the following tested xenobiotics: TC and MIN (Table 1). The xenobiotics inhibit the chlorophyll biosynthesis pathway [54,55] and limit photosynthesis, thus reducing the yield, hence the supposition that the inhibition of the plant growth rate is the result of chlorophyll degradation at the photosynthetic apparatus level. In the present study, the leaves exposed to the drugs were not always green, as the yellow pigment of TC and MIN were transferred to the tissues. Drugs, as an environmental stressor, cause a decrease in the amount of chlorophyll, but to increase stress resistance, they stimulate the biosynthesis of carotene. *Lemna aequinoctialis* exposed to streptomycins shows a decrease in the total amount of chlorophyll, with a simultaneous increase in the number of carotenoids [49]. However, in our study, different concentrations of antibiotics led to a reduction in the amount of TCC, and this reduction was more pronounced in the case of MIN than in TC (Figure 3C). While PQ did not respond to the tested drugs, the Fv/Fm index responded with a reduction of 33.7% and 44.3% on 19.2 mg × L^−1^ TC and MIN. This type of plant response may be the result of the reduction of active photosynthetic reaction centers and the simultaneous inhibition of electron transport in the photosynthetic system II (PS II) [56]. Meanwhile, the study conducted on thale cress (*Arabidopsis thaliana*), a commonly used model organism in biological research, revealed that the application of TC did not trigger a chloroplast-specific stress response. This was evidenced by the unaltered expression of chloroplast-specific chaperones, namely, PsaB and PsbA. However, tetracycline exhibited phytotoxic effects by inducing the expression of mitochondrial stress genes, disrupting mitochondrial translation, and inhibiting mitochondrial function. Since mitochondria are responsible for generating the majority of the cell’s ATP supply, this impairment can have significant consequences for cellular energy metabolism [54].

The compromised, induced by the tested TCs’ morphological plants’ parameters encompassed a spectrum of observable changes, including but not limited to variations in growth and biomass yield, leaf morphology, and quality of tissues. As is known, external changes and morphological features are only secondary effects caused by drug toxicity. These changes, which are often of an acclimatization nature, are a consequence of the plants’ responses at the physiological and cellular levels. As per Yang et al. [57], tetracycline and chlortetracycline exhibited varying degrees of inhibition on lettuce (*Lactuca sativa* L.) growth. Chlortetracycline demonstrated the following dual impact on plant growth: low concentrations facilitated growth, while high concentrations hindered both growth and photosynthesis. As research shows, significant changes in the physiological characteristics of plants occur when tissues absorb drugs from the environment. The pools of absorbed substances are also increased by natural plant protectants. *L. minor* not only accumulates tetracycline but also biogenic amines such as tyramine, putrescine, cadaverine, spermidine, and spermine, with spermine and tyramine showing heightened sensitivity upon tetracycline exposure [8]. Also, glyphosate exposure, for instance, triggers the accumulation of biogenic amines, primarily through the augmented activity of tyrosine decarboxylase and ornithine decarboxylase, enzymes within the biogenic amine biosynthetic pathway. Disturbances in homeostasis prompt changes in antioxidant enzyme activity; glyphosate exposure increases peroxidase activity [52], while exposure to silver nitrate leads to elevated catalase activity and malondialdehyde content, with variations observed in morphological parameters between *L. minor* and *Lemna minuta* [58]. Tetracycline was the most readily absorbed among the tested antibiotics. Optimal conditions for removing TC by *Lemna gibba* were determined through experimentation. A contact time of 6 days and a biomass of 11.4 g provided sufficient interaction and metabolic capacity for uptake and degradation. A TC concentration of 13.4 mg × L^−1^ was effectively managed by the plant without excessive stress or toxicity. Under these conditions, *L. gibba* significantly removed TC from the aquatic environment, highlighting its potential [48]. The biosorption of MIN was somewhat lower (Figure 5). It was proven that changes in morphological and physiological characteristics did not always occur simultaneously with the uptake of MIN by duckweed (Figure 1, Figure 2, Figure 3, Figure 4 and Figure 5). Therefore, either MIN was phototoxic even at the lowest tested concentrations, or this negative effect was the result of a high accumulation of large amounts of ions in the environment caused by the presence of the drug. Meanwhile, among the quinolones, nalidixic acid exhibited the highest absorption levels, while within the fluoroquinolone group, moxifloxacin, levofloxacin, and pefloxacin were comparatively less effectively absorbed by common duckweed. Notably, common duckweed employs an active biosorption mechanism rather than passive absorption (utilizing dead biomass) to remove quinolones from the solution. Moreover, it possesses the capability to transform the accumulated pollutants through internal processes, thereby contributing to the reduction of their concentrations in the environment [26]. Phytoremediation is a biological technology that participates in the removal of harmful pollutants from water and soil; however, phytoremediation research requires a comprehensive understanding of the processes and mechanisms [59]. As elucidated by Gadd [60], biosorption represents a distinct phenomenon operating autonomously from physicochemical metabolism, wherein substances are extracted from a solution by biological entities. This process entails the interplay between a solid phase, termed the biosorbent, and a liquid phase, typically a water-based solvent, harboring dissolved or suspended substances targeted for sorption, referred to as the sorbate [38]. Meanwhile, as reported by Maldonado et al. [61], pharmaceuticals are metabolized. The metabolization of toxic compounds in plants occurs in three phases. In phase I, toxic compounds are absorbed and converted into more reactive molecules, often through the action of enzymes such as cytochrome P450 monooxygenases, which can generate reactive oxygen species (ROS). Phase II involves the conjugation of these reactive molecules with hydrophilic entities like glutathione, facilitated by enzymes such as glutathione S-transferase, to neutralize and minimize their toxicity. In phase III, the detoxified compounds are sequestered and stored in vacuoles, apoplasts, or cell walls. Through this multi-phase process, plants effectively remove and neutralize toxic compounds [61]. Plants’ biological materials exhibit a notable affinity for both inorganic and organic contaminants, underscoring their versatility in absorbing diverse substances, as noted by Gadd [60]. Additionally, it would be ideal if the selected biomaterials should possess widespread availability, abundance in natural settings, and ease of cultivation [62]. Common duckweed emerges as a promising candidate in this context, fulfilling the aforementioned criteria as an indicator plant [39,42]. Consequently, identifying the most efficient, readily accessible, and cost-effective biomaterials constitutes a paramount challenge in biosorption research.

## 4. Materials and Methods

### 4.1. Plant Biosorbent

*L. minor* plants used in this study were obtained from the collection of the Department of Chemistry of the University of Warmia and Mazury in Olsztyn, Poland.

### 4.2. Chemical Adsorbates

Antibiotics:

Tetracycline hydrochloride and minocycline hydrochloride were purchased from Sigma Aldrich, St. Louis, MI, USA (Table 3).

### 4.3. Lemna Test

*L. minor* was grown in 50 mL of OECD medium for testing chemicals [42] in a plant growth chamber (ALL–Round–Al 185–4, Gent, Belgium) illuminated with fluorescent light (140 μmol photon m^−2^ × s^−1^ PAR) in a 16 h light/8 h dark cycle (mean maximum temperature of 20 °C during the daytime and 16 °C during the nighttime) for 7 days. All solutions were prepared using deionized water (Adrona Crystal 5 Basic water purification system, Riga, Latvia) with analytically pure TC and MIN. The responses of common duckweed to all concentrations of the tested solutions (0; 0.3; 0.6; 1.2; 2.4; 4.8; 9.6; and 19.2 mg × L^−1^) were determined based on the percent inhibition of growth rate (Ir), the percent reduction in yield (Iy), Chl *a* and Chl *b*, TCC, PQ of tissues, Fv/Fm, and the fresh weight (FW) and dry matter content (DM). The frond area was measured using the Lucia 5.0 program (Laboratory Imaging, s.r.o., Prague, Czech Republic). The following formulas were applied to calculate the values of Ir and Iy based on the number of duckweed fronds and frond area according to OECD, 2006 guidelines [40]:

*μ_i–j_* = [ln(*N_j_*) − ln(*N_i_*)]/*t* (1)

Ir = (*μ_c_* − *μ_T_*)/*μ_c_* × 100 (2)

Iy = (*b_c_* − *b_T_*)/*b_c_* × 100 (3)

where

*μ_i–j_*—average specific growth rate in time *i* to *j*;

*N_i_*—measurement variable in the test or control vessel at time *i*;

*N_j_*—measurement variable in the test or control vessel at time *j*;

*t*—period from *i* to *j*;

*μ_c_*—mean value of *µ* in the control group;

*μ_T_*—mean value of *µ* in the treatment group;

*b_c_*—final number of duckweed fronds and frond area minus the initial number of duckweed fronds and frond area in the control group;

*b_T_*—final number of duckweed fronds and frond area minus the initial number of duckweed fronds and frond area in the treatment group.

### 4.4. Chlorophyll, Total Carotenoid Content, and Phaeophytinization Quotient

The content of Chl *a*, Chl *b*, and TCC were determined in leaf extracts prepared with 96% (*v*/*v*) aqueous ethanol and centrifuged at 12,000× *g* for 15 min. The supernatant was separated; the pigments were quantified spectrophotometrically (Hitachi U-1800 spectrophotometer, Tokyo, Japan) according to [63], and the PQ was calculated according to [64]. The following formulas were used to calculate the concentrations of Chl *a* and Chl *b*, TCC, and PQ:

Chl *a = C*_Chl *a*_ × *V_e_* × *x*/*m* (4)

Chl *b = C*_Chl *b*_ × *V_e_* × *x*/*m* (5)

*C*_Chl *a*_ = 13.95 × *A*_665_ − 6.88 × *A*_649_ (6)

*C*_Chl *b*_ = 24.96 × *A*_649_ − 7.32 × *A*_665_ (7)

TCC = [1000 × *A*_470_ − 2.05 × Chl *a* − 114.8 × Chl *b*]/245 (8)

PQ = *A*_435_/*A*_415_ (9)

where

Chl *a*, Chl *b*—chlorophyll content in plant material, µg × mg^−1^;

*V_e_*—volume of ethanol extract, mL;

*x*—dilution coefficient;

*m*—sample weight, mg;

*C*_Chl *a*—_concentration of Chl *a* in the extract, µg × mL^−1^;

*C*_Chl *b*—_concentration of Chl *b* in the extract, µg × mL^−1^;

*A_i_*—solution absorbance at *i*-th wavelength.

### 4.5. Chlorophyll Fluorescence

The Fv/Fm of PSII (Photosystem II) was measured with the HandyPEA chlorophyll fluorescence system (Hansatech Instruments Ltd., Pentney, UK). Common duckweed leaves were placed in a leaf clip and stored in the dark for 30 min to quench chlorophyll fluorescence. After dark adaptation, chlorophyll was excited at a light intensity of 2500 [µmol × m^−2^ × s^−1^], and minimum chlorophyll fluorescence (Fo), maximum chlorophyll fluorescence (Fm) and variable chlorophyll fluorescence (Fv = Fm − Fo) were determined. The Fv/Fm of PSII was determined based on the chlorophyll fluorescence kinetics of common duckweed.

### 4.6. Antibiotic Biosorption

#### 4.6.1. Spectrophotometric Measurements

The extinction (*E*) of adsorbate solutions was measured with the Hitachi UV/VIS U-1800 spectrophotometer (Hitachi, Tokyo, Japan). The absorption spectra of TCs were set at 191–800 mm to determine the maximum absorption (*λ_max_*) of the analyzed solutions, with a concentration of 1.6 mg × L^−1^ each. The *λ_max_* values for quantitative measurements were determined at 370 nm for TC and 270 nm for MIN.

#### 4.6.2. Biosorption Measurements

The calibration curves of absorption vs. concentration *E* = *f*(*c*) were plotted for each adsorbate solution with the use of the previously determined *λ_max_* values. The Bs process was analyzed by placing 100 duckweed plants in 50 cm^3^ of adsorbate solution and stirring flask contents manually only once. The extinction of the analyzed solutions was determined before placing the plants in the solution (*E*_0_) and after 168 h (*E*_7_) using the Hitachi UV/VIS U-1800 spectrophotometer (Hitachi, Tokyo, Japan). After the measurement, 2 mL of the adsorbate solution was returned to the flask. The difference (*C*_0_ − *C*_7_) was the adsorption of a given adsorbate on the analyzed adsorbent.

Biosorption was calculated with the following formula:

*Bs* = (*C*_0_ − *C*_7_)/*m* × *V*/1000 (10)

where

*Bs*—biosorption [mg × g^−1^];

*C*_0_—initial concentration of adsorbate [mg × L^−1^];

*C*_7_—concentration of adsorbate after 168 h [mg × L^−1^];

*V*—solution volume [mL];

*m*—adsorbent mass [mg].

### 4.7. Statistical Analysis

The experiment was conducted in six replicates. The results were expressed as means ± standard deviation (SD). Data were processed statistically by two-way analysis of variance—ANOVA (F test) (Table 1). The experimental factors were the type and concentration of the applied antibiotic. Significant differences were determined using Tukey’s test at *p* < 0.01. The results of the experiment (Ir, Iy, FM, and DM of 100 duckweed plants, Chl *a* and Chl *b*, TCC, PQ, Fv/Fm, and the Bs of common duckweed) were processed in the STATISTICA 13.3 statistical package (TIBCO Software Inc., Palo Alto, CA, USA). Effective concentrations (EC_x_) were analyzed with a selected regression model to calculate the concentrations at 20% and 50% response levels.

## 5. Conclusions

Water pollution is a major global problem that requires the continuous assessment and control of water resources at all levels. This control preferably includes not only the assessment of the toxicity of xenobiotics but also methods of their elimination from the environment. This study examined how different concentrations of TC and MIN affected the growth, biomass, and photosynthetic parameters of common duckweed. The biosorption of these drugs by the duckweed triggered a series of negative impacts on the plant’s morphology and physiological processes. Significant increases in growth rates (Ir and Iy) were observed at higher concentrations of both antibiotics, with MIN showing greater toxicity than TC. The FM decreased significantly at higher concentrations, with TC more toxic than MIN. The DM increased, indicating tissue dehydration, with MIN showing higher phytotoxicity. Both antibiotics significantly reduced Chl *a*, Chl *b*, and TCC, with MIN being more toxic. The Fv/Fm ratio decreased significantly at the highest concentrations of both TCs. This study’s proposed water/wastewater treatment method was based on the biological method. The TC was absorbed more readily than MIN by duckweed, with Bs increasing proportionally to concentration. The biosorption-mediated compromise of morphological and physiological parameters underscores the intricate vulnerability of common duckweed to environmental contaminants and highlights the necessity for comprehensive assessments in elucidating the ecological ramifications of pharmaceutical pollution on aquatic ecosystems. As indicated, the consequences of the influence of TC on plants were slightly smaller in comparison to MIN, while the plants could biosorb this drug, even at the lowest tested concentration. This has shown that even a method of treating water and wastewater with drugs based on plant biosorption can be not only a standalone technique for water and wastewater management but also complementary to the systems used in it.

## Figures and Tables

**Table 1:** Analysis of variance (ANOVA) of the morphological and biochemical parameters (and indices) of common duckweed (*L. minor* L.) exposed to different concentrations (0–19.2 mg × L^−1^) of tetracycline hydrochloride (TC) and minocycline hydrochloride (MIN).

SoV	Ir	Iy	FM	DM	Chl *a*	Chl *b*	TCC	PQ	Fv/Fm	Bs
F-value										
Intercept	107.78 *	13,485 *	62,396.31 *	12,574.18 *	33,592.27 *	37,794.55 *	39,283.75 *	12,559,803 *	108,103.88 *	1856.19 *
Antibiotic (A)	1.98	2.89	44.16 *	10.39 *	32.09 *	35.02 *	28.87 *	7.16 *	19.38 *	164.58 *
Concentration (C)	12.87 *	15.49 *	29.14 *	3.07 *	58.01 *	56.98 *	52.69 *	8.04 *	11.51 *	279.28 *
A × C	0.56	0.42	3.04 *	0.81	6.13 *	8.85 *	5.65 *	7.17 *	1.23	54.77 *

SoV—a source of variation, A—type of antibiotic, C—concentration of antibiotic, A × C—antibiotic × concentration interactions, * significant at *p* < 0.01.

**Table 2:** The effect of tetracycline hydrochloride (TC) and minocycline hydrochloride (MIN) on the following plant parameters: percent inhibition of growth rate (Ir), percent reduction in yield (Iy), chlorophyll *a* (Chl *a*) and *b* (Chl *b*), total carotenoid content (TCC), phaeophytinization quotient (PQ) of tissues, maximum quantum efficiency (Fv/Fm), fresh mass (FM) and dry matter content (DM) of 100 duckweed plants.

Antibiotic	Parameter	Effective Concentration, mg × L^−1^	
		EC_20_	EC_50_
TC	Ir	0.73	5.69
	Iy	0.65	1.22
	Mean Ir and Iy	0.69	3.46
	FM	2.41	-
	DM	15.43	-
	Chl *a*	0.67	-
	Chl *b*	0.63	-
	TCC	15.78	-
	PQ	-	-
	Fv/Fm	16.18	-
MIN	Ir	0.26	3.45
	Iy	0.17	2.14
	Mean Ir and Iy	0.22	2.78
	FM	11.43	-
	DM	4.18	-
	Chl *a*	0.60	12.83
	Chl *b*	0.57	14.10
	TCC	2.43	18.15
	PQ	-	-
	Fv/Fm	13.89	-

**Table 3:** Characteristics of the TCs used in the experiment.

Chemical Compound	Structural Formula	Empirical Formula	CAS Number	Molecular Weight [g × mol^−1^]	Form/Color
Tetracycline hydrochloride (TC)	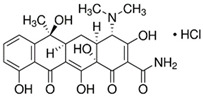	C_22_H_24_N_2_O_8_ · HCl	64-75-5	480.90	powder/ yellow
Minocycline hydrochloride (MIN)	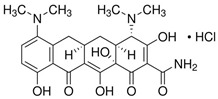	C_23_H_27_N_3_O_7_ · HCl	13614-98-7	493.94	powder/ yellow

## Data Availability

Data are contained within the article.

## Abbreviations

TCs	tetracyclines
TC	tetracycline
MIN	minocycline
FM	fresh mass
DM	dry matter content
DW	dry weight
Ir	percent inhibition of growth rate
Iy	percent reduction in yield
Chl *a*	chlorophyll *a*
Chl *b*	chlorophyll *b*
TCC	total carotenoid content
PQ	phaeophytinization quotient
Fv/Fm	maximum quantum efficiency
EC_x_	effective concentration associated with x% response (20% and 50%)
Bs	antibiotic biosorption

## Appendix A

**Table A1:** The Standard deviation (±SD) of the morphological and biochemical parameters (and indices) of common duckweed (*L. minor* L.) exposed to different concentrations (0–19.2 mg × L^−1^) of tetracycline hydrochloride (TC) and minocycline hydrochloride (MIN).

Feature	Tetracycline Hydrochloride, mg × L^−1^							
	0.00	0.30	0.60	1.20	2.40	4.80	9.66	19.20
±SD for Percent inhibition of growth rate (Ir)	-	7.07	14.35	20.26	17.68	20.52	11.68	15.18
±SD for Percent inhibition in yield (Iy)	-	12.42	14.81	20.32	11.56	18.81	20.66	26.86
±SD for Fresh mass (FM)	4.74	18.52	1.20	11.32	4.41	6.15	6.71	8.72
±SD for Dry matter content (DM)	1.48	1.76	1.75	1.85	2.27	2.12	1.40	1.82
±SD for Chlorophyll a content (Chl *a*)	0.03	0.06	0.06	0.02	0.00	0.01	0.02	0.02
±SD for Chlorophyll b content (Chl *b*)	0.02	0.03	0.02	0.01	0.04	0.01	0.01	0.01
±SD for Total carotenoid content (TCC)	0.01	0.02	0.02	0.01	0.03	0.01	0.01	0.01
±SD for Phaeophytinization quotient (PQ)	0.00	0.00	0.00	0.00	0.00	0.00	0.00	0.00
±SD for Maximum quantum efficiency (Fv/Fm)	0.02	0.01	0.02	0.02	0.02	0.03	0.03	0.04
±SD for Antibiotic biosorption (Bs)	-	0.05	0.03	0.04	0.10	0.19	0.66	0.85
**Feature**	**Minocycline hydrochloride, mg × L^−1^**							
	**0.00**	**0.30**	**0.60**	**1.20**	**2.40**	**4.80**	**9.66**	**19.20**
±SD for Percent inhibition of growth rate (Ir)	-	12.63	8.45	17.77	6.91	11.42	10.31	20.62
±SD for Percent inhibition in yield (Iy)	-	12.06	9.14	16.12	11.56	21.43	20.66	41.32
±SD for Fresh mass (FM)	4.74	19.78	4.41	16.00	4.74	30.30	6.86	8.91
±SD for Dry matter content (DM)	6.35	7.20	7.23	7.36	7.55	7.76	7.65	9.94
±SD for Chlorophyll a content (Chl *a*)	0.13	0.17	0.06	0.06	0.05	0.04	0.03	0.04
±SD for Chlorophyll b content (Chl *b*)	0.05	0.07	0.02	0.03	0.02	0.01	0.01	0.01
±SD for Total carotenoid content (TCC)	0.04	0.06	0.02	0.02	0.02	0.01	0.01	0.01
±SD for Phaeophytinization quotient (PQ)	0.00	0.00	0.00	0.00	0.00	0.00	0.00	0.00
±SD for Maximum quantum efficiency (Fv/Fm)	0.01	0.04	0.02	0.02	0.08	0.06	0.03	0.04
±SD for Antibiotic biosorption (Bs)	-	0.00	0.06	0.25	1.51	0.76	0.98	0.00
